# Acquired Jugular Vein Aneurysm

**DOI:** 10.1155/2009/535617

**Published:** 2009-02-25

**Authors:** Erkki Hopsu, Jussi Tarkkanen, Seija I. Vento, Anne Pitkäranta

**Affiliations:** ^1^Department of Otorhinolaryngology, Kymenlaakso Central Hospital, 48210 Kotka, Finland; ^2^Helsinki University Central Hospital Laboratory (HUSLAB), Department of Pathology, Haartman Institute, University of Helsinki, 00014 Helsinki, Finland; ^3^Department of Otorhinolaryngology, Helsinki University Central Hospital, 00290 Helsinki, Finland

## Abstract

Venous malformations of the jugular veins are rare findings. Aneurysms and phlebectasias are the lesions most often reported. We report on an adult patient with an abruptly appearing large tumorous mass on the left side of the neck identified as a jugular vein aneurysm. Upon clinical examination with ultrasound, a lateral neck cyst was primarily suspected. Surgery revealed a saccular aneurysm in intimate connection with the internal jugular vein. Histology showed an organized hematoma inside the aneurysmal sac, which had a focally thinned muscular layer. The terminology and the treatment guidelines of venous dilatation lesions are discussed. For phlebectasias, conservative treatment is usually recommended, whereas for saccular aneurysms, surgical resection is the treatment of choice. While an exact classification based on etiology and pathophysiology is not possible, a more uniform taxonomy would clarify the guidelines for different therapeutic modalities for venous dilatation lesions.

## 1. Introduction

**Figure 1:**
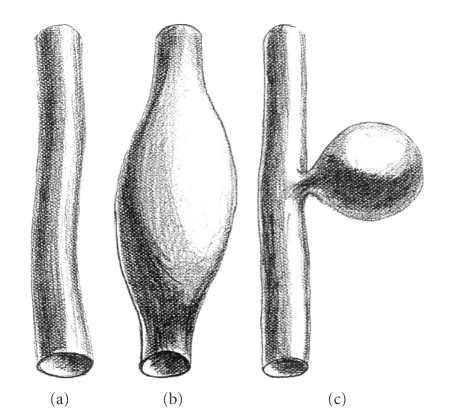
Schematic drawing of (a) a normal vein, (b) a phlebectasia, and (c) an aneurysm (courtesy of Seppo Piirainen, Peijas-Rekola Hospital, Vantaa).

The most common lesions of venous malformations in the head and neck region are internal jugular vein aneurysms and phlebectasias. Although these terms are frequently used as synonyms, they pertain to two divergent conditions, likely of different etiology and pathophysiology. Macroscopically, aneurysms are described as saccular [1, 2] and phlebectasias as fusiform [1, 3] (Figure 1).

While phlebectasia in the neck region is considered to be congenital in origin or to arise from a primary congenital weakness within the muscular layer of the venous wall [4–6], venous aneurysm often occurs secondary to trauma or in association with diseases involving veins [2]. Fusiform jugular phlebectasia is most often described as manifesting in childhood [7]. Secondary or acquired venous dilatation lesions are usually saccular in form, and multiple etiological factors have been suggested for these thrombosed venous aneurysms, which are typically seen in adults [2, 8].

We report on an adult patient with a large tumorous mass on the left side of the neck identified as a jugular aneurysm.

## 2. Report of a Case

Three days prior to admission to the University Central Hospital in Helsinki, a 71-year-old woman noticed a rapidly growing lump on the left side of her neck. Physical examination revealed a mass underneath the anterior border of the sternocleidomastoid muscle in the supraomohyoid region of the neck. The tumor was slightly tender and warm upon palpation, but the patient had no infectious symptoms. There was no known trauma to this area. Ear, nose, and throat status was normal. C-reactive protein and blood white cell count were normal. Past medical history included elevated blood pressure and bronchial asthma with medication. No previous operations or arterial/venous catheterizations had been performed.

Ultrasound revealed a smooth, rounded cystic configuration (diameter 3.5 × 1.9 cm) filled with a thick fluid. In differential diagnosis, either an infected cyst or a necrotized lymph node was suspected.

The patient was treated with intravenous kefuroxime and metronidazole for one day and thereafter with per oral kefalexine and metronidazole for ten days because of a suspected infected lateral neck cyst. During a six week follow-up, the mass had not regressed. A malignant involvement could not be excluded and the surgery was performed.

**Figure 2:**
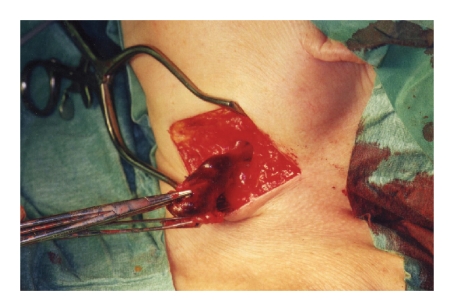
The jugular vein is twisted as the aneurysm is pulled up before resection.

Upon surgery under general anesthesia, a saccular venous aneurysm was detected. A short, wide-based connection was present between the venous wall of the jugular vein and the cyst-like aneurysm, which had a smooth and rounded contour. The pathological segment of the jugular vein was resected and the free ends of the vessel ligated (Figure 2). The patient recovered well.

**Figure 3:**
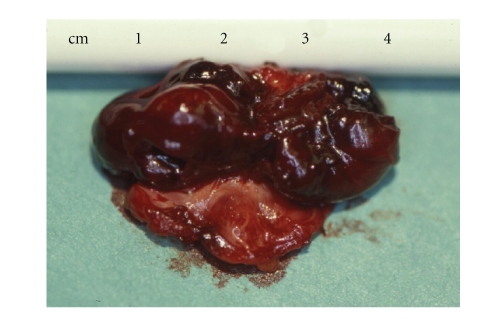
Macroscopic view of the removed aneurysm cut in half longitudinally.

**Figure 4:**
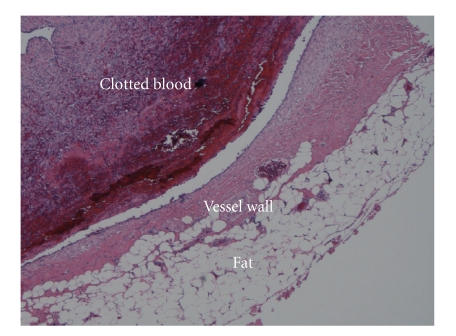
Microscopic view of the aneurysm with an organized blood clot in the lumen and a focally thinned muscular wall.

Pathological examination showed a mass of 3.5 × 2.0 × 1.8 cm in diameter with a macroscopically normal venous wall encapsulating an organizing hematoma inside the lumen (Figure 3). Fibrotic tissue and fat were observed outside the venous wall. Histologically, an organized hematoma was seen inside a venous lumen, which had a focally thinned muscular layer. No signs of infection or neoplasia were present in the tissue specimen (Figure 4).

## 3. Comment

No established classification of venous malformations can be found in vascular pathology handbooks, and the terms used for venous dilatation lesions vary. Two of these terms—aneurysm and phlebectasia—can be differentiated based on macroscopic clinicopathological findings of the lesions [1]. The pathogenesis of venous aneurysms and phlebectasias remains, however, obscure, with scattered pathological findings and conflicting theories of pathophysiology [5, 7].

One of the first descriptions of an internal jugular phlebectasia by Gerwig Jr. [3] defines phlebectasia as a solitary fusiform dilation of a vein. Abbott and Leigh [9], in categorizing aneurysmal venous diseases, made a distinction between congenital primary fusiform and saccular lesions, suggesting that a saccular lesion can be classified as a true venous aneurysm. Eifert et al. [1] also made a distinction between the terms phlebectasia and aneurysm.

Opinions on the etiology of aneurysms and phlebectasias vary. Jugular fusiform phlebectasia is thought to be a childhood disease [4, 7, 10–12]. In these phlebectasias, patent blood flow is typically present [11–13] and the size of the internal jugular phlebectasia can even become less evident as the child reaches puberty [10]. In localized phlebectasias, the tendency for thrombus formation is not higher than in normal veins [1, 6]. Saccular aneurysms, by contrast, have a predilection for thrombosis [2, 8]. The etiopathology of thrombosed aneurysms appears to differ from that of childhood jugular vein phlebectasias. The macroscopic appearance of these lesions (fusiform versus saccular) seems to correlate with the age of the patient. However, saccular lesions do occasionally present in children [14] and fusiform jugular lesions, phlebectasias, have been reported in adults [13], especially in the anterior or external jugular veins [15].

Pathological changes of vein walls vary histologically [2, 5, 8]. In phlebectasias, the muscular layer is usually thinned. In saccular aneurysms, which tend to thrombose, degenerative histological changes are seen [2, 8]. Based on histological findings, aneurysm is considered a more apt term than phlebectasia when all layers of the vein wall are present [16].

Inconsistency in the terms used is reflected in the nonuniform recommendations for treatment strategies of venous dilation lesions. The outcome of venous dilatation lesions is dependent on their anatomical location [5, 17]. Thus, treatment options, besides being affected by the form of the lesion, also depend on its location. Lesions in the head and neck region, including the internal jugular vein, tend to have a benign natural history, with no serious complications being reported [5, 18]. In rare instances, an embolism or rupture of a venous aneurysm may occur in other locations, resulting in a surgical emergency or death, especially in the case of deep aneurysms of the abdominal region and the lower extremities [5, 17, 18]. Although no reports of life-threatening complications for jugular vein aneurysms exist, a surgical resection is the treatment of choice for saccular aneurysms in the jugular vein [8, 14, 16]. For jugular phlebectasias, by contrast, conservative follow-up is preferred [4, 6, 7, 10–13, 15], with excision only if the lesion is symptomatic, enlarging, or disfiguring [6, 7, 17].

Jugular venous aneurysms may be clinically confused with branchial cysts, laryngoceles, hemangiomas, and lymphangiomas, or other cystic lesions, such as carcinoma arising in primary lateral neck cyst or metastatic squamous cell carcinoma. Ultrasonography can assist in making the correct diagnosis but was not useful in this case. Doppler sonography, computed tomography, or angiographies are helpful investigations to increase accuracy in diagnosis of a cervical mass [19]. Gadolinium-enhanced magnetic resonance angiography imaging is a reliable method for neck vein imaging [20].

The etiopathology of the aneurysm here remains totally open, its manifestation appeared to be spontaneous and acquired, and histological examination yielded no special pathological clues of its etiology.

## 4. Conclusions

The rare presentation and inconsistent terminology of venous dilatation lesions can cause confusion in choosing an optimal treatment. The treatment options depend on the location and the form of the lesion. For jugular phlebectasias, conservative treatment is usually recommended, whereas for saccular aneurysms, which often present with thrombosis, surgical resection is the treatment of choice. Although an exact taxonomy based on etiology or pathophysiology is not yet possible, a more uniform nomenclature would clarify the guidelines for different therapeutic modalities for venous dilatation lesions.
